# Metabolic glycoengineering enhances stem cell therapy for cardiac arrest-induced brain injury: A new dimension in neuroregenerative medicine

**DOI:** 10.1016/j.jointm.2025.12.006

**Published:** 2026-02-14

**Authors:** Xiao Liu, Xiaofeng Jia

**Affiliations:** 1 Department of Neurosurgery, University of Maryland School of Medicine, Baltimore, MD, USA; 2 Department of Orthopedics, University of Maryland School of Medicine, Baltimore, MD, USA; 3 Department of Anatomy and Neurobiology, University of Maryland School of Medicine, Baltimore, MD, USA; 4 Fischell Department of Bioengineering, University of Maryland, College Park, MD, USA

Cardiac arrest (CA) often results in global cerebral ischemia and long-term neurological disability, highlighting the need for therapies that extend beyond supportive care. Neural stem cell (NSC)-based approaches offer a potential regenerative strategy,^[^^1^^]^ yet their clinical translation has been limited by poor cell survival and inadequate functional integration into injured neural circuits. Metabolic glycoengineering (MGE) provides a non-genetic strategy to enhance the therapeutic capacity of NSCs.^[^^2^^]^ This preclinical work^[^^2^^]^ using the thiol-modified sugar analog Ac5ManNTProp (TProp) demonstrates that MGE-treated NSCs exhibit improved survival and neuronal differentiation in vitro. In a rat CA model, intranasally delivered TProp-modified NSCs resulted in better hippocampal neuron preservation, enhanced synaptic repair, and improved neurologic recovery compared with unmodified NSCs. This editorial summarizes and contextualizes these findings, highlighting that MGE-enhanced NSC therapy offers a compelling new avenue for clinical translation and has the potential to advance regenerative therapies for CA-induced brain injury in neurocritical care.

## Stem Cell Therapy in CA: Progress and Limitations

CA remains a pressing clinical challenge, with most survivors experiencing long-term neurological impairment due to global cerebral ischemia. Although advances in resuscitation and post-CA care have improved survival rates modestly, the restoration of meaningful neurological function remains limited. Existing neuroprotective interventions have yielded inconsistent and limited clinical benefits, underscoring the need for new therapeutic strategies aimed at repairing ischemic brain injury.

Stem cell-based therapy has emerged as a promising approach due to the capacity of NSCs to differentiate into functional neurons, secrete neurotrophic factors, and modulate neuroinflammation.^[^^1^^]^ However, the translation has been hindered by intrinsic biological barriers, including poor post-transplant cell survival, inadequate retention within injured regions, and limited neuronal differentiation in the hostile ischemic microenvironment.^[^^2^^]^ These barriers are particularly pronounced following CA, where widespread neuronal loss and inflammatory activation create a hostile environment for cell engraftment.

For stem cell-based therapies, MGE offers distinct advantages by improving homing and retention in target tissues,^[^^3^^]^ modulating key processes such as cell survival, differentiation, and signaling,^[^^3^^]^ and enabling highly specific surface functionalization of cells or extracellular vesicles without genetic manipulation.^[^^4^^]^ Our recently published study^[^^2^^]^ addresses these limitations by applying MGE to modify NSCs before transplantation. In this work, NSCs were treated with Ac5ManNTProp (TProp), a thiol-modified sugar analog that is metabolically incorporated into cell-surface sialoglycans.^[^^5^^]^ This modification improves the cell viability, activates the Wnt/β-catenin signaling pathway, and promotes neuronal differentiation. When delivered intranasally and intracerebroventricularly following CA in a rat model, these metabolically glycoengineered NSCs significantly improved neurological recovery, neuronal preservation, and synaptic repair compared to naive NSC therapy.^[^^2^^]^

Our study^[^^2^^]^ builds on earlier work demonstrating that intranasally delivered naive NSCs can migrate to the hippocampus and provide neuroprotection after CA-induced ischemic brain injury.^[^^6^^]^ The new findings suggest that improving the intrinsic biological state of NSCs before transplantation might be as important as selecting the correct delivery route or timing.

## Mechanistic Insight: Wnt Signaling and Synaptic Repair

Glycosylation is a fundamental regulator of cell–cell communication, receptor availability, and intracellular signaling.^[^^7^^]^ In the context of MGE, incorporating thiol-modified sugars such as TProp reshapes the surface glycan architecture of NSCs. This targeted remodeling facilitates more effective ligand–receptor interactions and stabilizes signaling complexes at the cell membrane, thereby enhancing pathways critical for neuronal regeneration and maturation. Among these pathways, the Wnt/β-catenin pathway appears as the most directly influenced and most relevant to the outcomes observed in this preclinical study.^[^^2^^]^

TProp-treated NSCs exhibit increased β-catenin accumulation and enhanced phosphorylation of GSK-3β, consistent with activation of Wnt signaling. Glycan modifications can improve the clustering or accessibility of Wnt receptors, promoting more efficient signal transduction.^[^^8^^]^ Activation of this pathway is well-established to support neuronal differentiation and synaptic maturation. Correspondingly, TProp-NSCs exhibited higher expression of neuronal markers in vitro and improved transplanted cell integration into hippocampal circuits *in vivo*.^[^^2^^]^

Importantly, the downstream effects extended to synaptic repair. Key synaptic proteins – synapsin1, VGLUT2, and PSD95 – were significantly restored in ischemia-vulnerable hippocampal regions following transplantation of MGE-modified NSCs.^[^^2^^]^ These findings reveal a coherent mechanistic sequence: MGE-induced glycan remodeling in NSCs enhances Wnt/β-catenin signaling, which drives neuronal differentiation and promotes the remodeling of functional synaptic networks essential for neurological recovery after CA.

## Intranasal Delivery: A Clinically Feasible Route

Intranasal delivery has emerged as a particularly attractive route for translational application because it bypasses the blood–brain barrier without requiring invasive neurosurgical procedures.^[^^1^^,^^9^^]^ Growing preclinical evidence supports its ability to deliver viable cells to deep brain regions, including the hippocampus,^[^^6^^]^ via the olfactory and trigeminal pathways.^[^^10^^]^ This is especially relevant for CA survivors, who are often medically unstable and poor candidates for invasive intracerebral injection. The intranasal route enables repeated dosing, rapid initiation of therapy, and bedside administration – features that align well with post-resuscitation care.

The preclinical study reinforces this translational potential for post-CA survivors by demonstrating that MGE-NSCs, when delivered intranasally, not only effectively reach ischemia-vulnerable hippocampal regions but also promote significant structural and functional recovery. These findings support intranasal administration as a potentially feasible, scalable, and clinically adaptable approach for treating CA-induced brain injury. Moreover, optimizing the intrinsic properties of NSCs can further amplify the benefits of this practical delivery route.

## Positioning Within the Current Landscape of Translational Neuroscience

Our study exemplifies a strategic shift in regenerative medicine, in which therapeutic cells are modified in advance to better withstand and respond to the post-ischemic environment, rather than relying solely on the endogenous capacity of the injured brain to support transplanted cells. Comparable logic has been applied in peripheral nerve and spinal cord injury research, where stem cell administration aims not only to replace lost cells but also to modulate glial activation and neuroinflammation.^[^[11], [12]^]^ The present findings extend this concept to global ischemic injury and provide molecular-level justification for pretreatment strategies prior to transplantation.

A key conceptual advance of this work lies in its distinction between traditional neuroprotection and true synaptic restoration. Traditional neuroprotective strategies focus on minimizing acute cell death or reducing inflammation, yet rarely address the deeper structural loss that underlies long-term cognitive and neurological deficits after global ischemia. In contrast, MGE-enhanced NSCs demonstrated the capacity not only to preserve neuronal populations but also to rebuild excitatory synaptic architecture. This shift, from preventing further injury to actively recovering and remodeling neural circuitry, positions the current approach within a new therapeutic paradigm in translational neuroscience. By optimizing the intrinsic biological state of NSCs before transplantation, the study moves beyond traditional supportive strategies and toward interventions capable of restoring functional neural networks. This distinction underscores the potential of MGE-enhanced cell therapy as a transformative step forward in regenerative treatment for global ischemic brain injury.

While the findings are promising, several challenges should be addressed before this strategy can be translated into clinical practice. The validation in large-animal or more complex systems would be beneficial to confirm cell migration, survival, and functional efficacy in brains that more closely resemble human anatomy. Furthermore, the optimal dosing strategy and therapeutic window remain unelucidated. Determining how soon after CA stem cell treatment would be initiated, how frequently NSCs should be administered, and what cell dose provides maximal benefit without undue risk are critical next steps. Additionally, the interaction between MGE-enhanced NSC therapy and current established standard-of-care interventions, including but not limited to targeted temperature management, has not yet been evaluated. Targeted temperature management and other post-arrest treatments may influence inflammation, metabolism, and cell signaling; thus, dedicated studies are needed to assess compatibility and potential synergistic benefits. Finally, practical considerations such as scalable manufacturing and regulatory compliance should be addressed before the clinical translation. Together, addressing these challenges, while building on the key preclinical findings of this study, offers valuable opportunities to advance toward more effective clinical translation.

## Conclusions

Our study provides important evidence that the therapeutic capacity of NSCs can be significantly enhanced through MGE, leading to improved neuronal survival, synaptic restoration, and functional recovery after CA.^[^^2^^]^ The work advances the field by demonstrating that cell-intrinsic engineering can overcome longstanding biological and clinical barriers that have limited stem cell therapy for global cerebral ischemia. More broadly, this approach integrates materials chemistry, stem cell biology, and resuscitation neuroscience, representing a significant step toward clinically viable neuroregenerative therapy after CA-induced ischemic brain injury. Comprehensive evaluation of safety and feasibility, optimal dosing for the MGE stem cell therapy, and compatibility with standard-of-care interventions are essential and will further move MGE-NSCs toward therapeutic applications to improve future clinical translation.

## CRediT authorship contribution statement

**Xiao Liu:** Writing – original draft. **Xiaofeng Jia:** Writing – review & editing.
